#   Issues facing the future health care workforce: the importance of demand modelling

**DOI:** 10.1186/1743-8462-6-12

**Published:** 2009-05-07

**Authors:** Leonie Segal, Tom Bolton

**Affiliations:** 1 Health Economics and Policy Group, Division of Health Sciences, University of South Australia, Adelaide, South Australia, Australia

## Abstract

This article examines issues facing the future health care workforce in Australia in light of factors such as population ageing. It has been argued that population ageing in Australia is affecting the supply of health care professionals as the health workforce ages and at the same time increasing the demand for health care services and the health care workforce.

However, the picture is not that simple. The health workforce market in Australia is influenced by a wide range of factors; on the demand side by increasing levels of income and wealth, emergence of new technologies, changing disease profiles, changing public health priorities and a focus on the prevention of chronic disease. While a strong correlation is observed between age and use of health care services (and thus health care workforce), this is mediated through illness, as typified by the consistent finding of higher health care costs in the months preceding death.

On the supply side, the health workforce is highly influenced by policy drivers; both national policies (eg funded education and training places) and local policies (eg work place-based retention policies). Population ageing and ageing of the health workforce is not a dominant influence. In recent years, the Australian health care workforce has grown in excess of overall workforce growth, despite an ageing health workforce. We also note that current levels of workforce supply compare favourably with many OECD countries. The future of the health workforce will be shaped by a number of complex interacting factors.

Market failure, a key feature of the market for health care services which is also observed in the health care labour market – means that imbalances between demand and supply can develop and persist, and suggests a role for health workforce planning to improve efficiency in the health services sector. Current approaches to health workforce planning, especially on the demand side, tend to be highly simplistic. These include historical allocation methods, such as the personnel-to-population ratios which are essentially circular in their rationale rather than evidence-based. This article highlights the importance of evidence-based demand modelling for those seeking to plan for the future Australian health care workforce. A model based on population health status and best practice protocols for health care is briefly outlined.

## Background

The challenges facing Australia's health workforce have been the subject of recent intense public interest, with perceptions of widespread and increasing workforce shortages that could impact upon patient care [1-4]. This mirrors concerns about health workforce shortages internationally, for example as expressed by the World Health Organization – although for WHO critical shortages are particularly identified with sub-Saharan Africa and Asia [5].

Perceptions of health workforce shortages are driven in part by concern by policy-makers of the impact of 'population ageing' upon the supply of and demand for health professionals [6,7]. A major demographic shift towards an older population has been occurring in the Australian population over several decades (the product of increasing life expectancy, lower birth rates and the ageing post-war baby boom generation). As this shift has been occurring, government inquiries have sought to understand how the change in the population age structure might impact on the capacity of the health workforce to deliver needed services.

The Productivity Commission in its 2005 report on Australia's health workforce, details shortages in the health workforce "in general practice, various medical specialty areas, dentistry, nursing and some key allied health areas." [6]. It suggested shortfalls of 800 to 1300 GPs in 2002 (~5% of the GP workforce), and an anticipated shortfall of 10–12,000 nurses (~5% of the nursing workforce) in 2006 and 12–13,000 in 2010; citing the 2004 Australian Health Workforce Advisory Committee (AHWAC) 2003–2004 annual report, [8] (This was in turn based on research by Access Economics (2004) [9], Preston [10], Shah and Burke [11] and Karmel and Li [12].) Other commentators have voiced similar concerns about existing and impending health workforce shortages [13]. However, the basis for these observations and predictions are highly simplistic models that fail to adjust for any of the complex influences on the demand for and supply of health workers, as described below. They thus fail to provide a sound basis for health workforce planning, which requires a sophisticated understanding of the main drivers of demand and supply. A factor recognised in the recent establishment of a National Health Workforce Taskforce through AHMAC (Australian Health Minister's Advisory Council) to take on a health workforce planning role [14] recognises the complexity of this task and the inadequacy of current approaches to health workforce planning.

In this paper we discuss some of the key drivers of health workforce demand and health workforce supply and conclude with a brief discussion of alternative approaches to modelling health workforce demand.

### Influences on the health workforce supply

The health care workforce incorporates a range of vocations operating in different industry settings; medical practitioners, nurses, allied health professionals (physiotherapists, dieticians, occupational therapists, optometrists, clinical psychologists, social workers, indigenous health workers etc.) and other occupations, working in hospitals and other institutional settings (eg residential care facilities) and community settings including private practice, community health services and non-health settings (such as schools and workplaces). There are over 450,000 paid health professionals in Australia, of whom over 50% are nurses, 12% medical practitioners and 9% allied health professionals [5,15,16].

**Figure 1:**
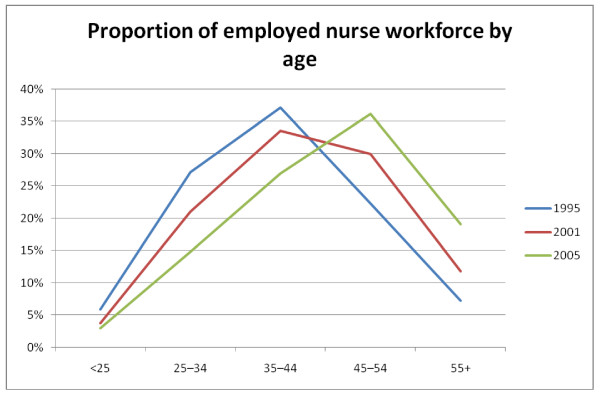
**The age structure of the Austra*lian nursing workforce 1995*, 2001, 2005 Source: AIHW **[17-20].

As noted the ageing of the population and the associated ageing of the health workforce as illustrated, for example, in Figure 1[17-20] for the Australian nursing workforce is postulated to be precipitating a crisis in heath workforce supply.

Commentators have cited the increasing proportion of health care workers reaching retirement age, the traditionally lower per-person hours worked with increasing age combined with feminisation of the medical workforce also associated with lower average hours per worker – as undermining workforce supply [6]. Exacerbated by increasing pressures in the work place, especially in public hospitals, that result in high rates of staff turnover and departures from the industry [21,22].

The major focus of the debate has been the ageing of the workforce and what is seen as an inevitable decline in numbers. However, drawing conclusions from cross-sectional data ignores the many contrary influences affecting health workforce supply. Participation rates at older ages and mean hours of work are increasing, especially amongst women. This is in part a direct response to policy, such as changes in superannuation rules and other regulatory changes specifically designed to delay retirement[23]. More generally, female participation rates in Australia are increasing across all age groups, related to delayed child rearing, smaller family size, policy changes in childcare and changes in community attitudes. This has seen female participation rates increase from 42.3% in Jan 1979 to 58.4% in March 2008 [23,24]. Projections undertaken by Evans & Keeley [25] suggested a dramatic increase in participation rates for older married women in the next two decades; for married women over 55 with children post-school age from 59% in 2002 to 89% by 2022. This is highly significant given the largely female health professional workforce. Healy [26] also argues that an older workforce may also have greater levels of skills and experience and potentially greater efficiency.

The supply and mix of health professionals through new entrants, training, changing roles and retention of staff has shown considerable responsiveness to direct policy initiatives as well as the indirect drivers of changing technology and service demands. This is seen in a constantly changing health workforce mix, a process detailed by Duckett [4]. Recent innovative changes to traditional health workforce roles in Australia (such as the expanding role of the practice nurse and nurse practitioner and the development of new para-professional roles) will allow an even greater responsiveness to future health workforce supply challenges[27]. The recent focus on chronic disease prevention through the Australian Better Health Initiative is seeing considerable discussion about new allied health roles, such as 'lifestyle coaches'; and the development of new courses to train for these community-based illness prevention roles [28,29].

The United Kingdom, for example, has increased the size of the health care workforce in a short time period, with an appropriately targeted policy mix; including new funding for additional consultants, GPs, and nurses within the NHS, an increase in medical school places and hospital-based reforms to improve the work environment [30]. These initiatives have seen in the UK National Health Service a growth in the number of GPs by 20.1%, of qualified nurses by 26.8% and of allied health professionals by 35.7% in the period 1997 to 2005 [30]. The health care workforce in the UK has demonstrated the potential effectiveness of policy levers. There is no reason to assume a similar range of policies would not be effective in Australia, especially given the strong competition for places in clinical schools [31]. The capacity for Australia's health workforce to expand, despite an ageing workforce is evident from a simple analysis of the available data. Census figures show a growth in the health workforce in Australia of over 3% per annum, significantly faster than the rate of population growth of 1.2% to 1.5% per annum 2000–2008) [32]. The AIHW [33] concurred reporting a 26% increase in the number of people employed in health occupations between 2000 and 2005, compared with an 18.04% rise in the workforce overall [24].

Health workplaces in Australia are responding to current and projected health workforce and other capacity problems with a range of reforms. Several successful initiatives are described in the Supplement to the MJA, [34]. Papers identify productivity gain and reduced workforce turnover through redesign of work processes. An example reported is Flinders Medical Centre in Adelaide, which employed a 'lean thinking' approach to reorganise care. Key principles were involvement of 'shop floor staff', a patient focus and detailed understanding of current work activities. Authors report an increase in productivity, eg 40% increase in ED throughput, over 70% reduction in serious adverse events and stabilisation of staffing "within the ED and across nursing services throughout the hospital" [35]

Another policy lever has been to use a range of initiatives to supplement the Australian health workforce with overseas trained doctors and nurses. The Australian Health Workforce Advisory Committee (AMWAC), estimated overseas trained doctors made up 25% of the Australian medical workforce in 2005, up from an estimated 19% in 1995; (Productivity Commission [6]). The AIHW 2005 medical labour force survey (covering all states except NSW and NT) also reports 78.0% of the health workforce trained in Australia, but with considerable variation across the country, (65.5.% WA). Overseas recruitment has been designed primarily to address regional maldistribution and a past failure to increase training places for health professionals. High competition from local students for training places suggests that a shift towards a higher local share is feasible, if resourced.

The evidence concerning health workforce supply suggests a picture of a health workforce growing, despite an ageing population and ageing workforce, and one that is highly responsive to policy levers. The challenge is to ensure the policy levers are driven by a sound understanding of health workforce demand. Establishing the desirable level and mix of the health workforce, which reflects the optimal mix of health services, needs to be a focus of health workforce research. Health workforce supply strategies can then be devised to meet underlying need.

### Health workforce demand

Rarely is a clear distinction made between 'expressed' health workforce demand and underlying need. Expressed workforce demand is essentially defined by funded places (essentially current supply plus unfilled positions), while the concept of underlying need refers to the health workforce (size and mix) that will meet societal health sector objectives (including equity) in an efficient manner. In the context of market failure as typifies the health sector and the health labour market[36,37], concepts of expressed demand and underlying need are likely to depart. In a health workforce planning exercise, it is the underlying health workforce need, which in turn reflects the need for health care – including health promotion – that should drive supply, not expressed demand. We also note that the latter concept being mediated by supply is not exogenous.

The Productivity Commission (p.18)[6] argued that the demand for health care services and consequently for the health care workforce will primarily be driven by the ' ageing of the population' and associated increasing disability rates, together with changing technology, changing burden of disease, higher incomes and expectations. The background papers to the Australian 2020 summit also presumes a dominant influence of an ageing population on demand for health care (and thus the health care workforce) [38].

However the evidence suggests that the role of ageing is highly uncertain, as the observed relationship between age and health care use and thus health workforce requirement is entirely mediated through illness. It simply is not correct to use cross-sectional data of health care costs by age, for projecting health care use and health care workforce demand. End-of-life events are consistently found to make a major contribution to health care costs, which, correlated with increasing age does not mean ageing is an independent determinant of health care costs. Calver, Bulsara and Boldy [39] in their study of hospital costs relating to end of life events in Western Australia concluded that "Older decedents were not more likely to be hospitalised than younger decedents in the final three years of life. Moreover, once hospitalised, their in-patient costs were lower." They cited a number of studies that reach a similar conclusion, including Gray [40] who reported that "the relationship between age and health expenditure was weak and possibly inverse once proximity to death was allowed for."

As noted by Calver and colleagues "Failure to account for proximity to death will overemphasise the impact of population ageing on health care expenditure, because older people have a higher probability of dying." International studies to develop risk adjusters for insurance premiums, also find that ageing as an independent variable accounts for less than 3% of variation in health care costs [41]. Risk adjustment models are increasingly incorporating health status as the primary drivers. Breyer and Felder [42] also warned against simply undertaking a cross-sectional analysis of hospital admission rates by age and applying this to future population projections, arguing that time before death is a better predictor. (See also Coory (p.581)[43].

Annual death data show that in contrast to (or in part because of) the ageing of the population (people are staying alive longer) the annual number of deaths in Australia is increasing at only a modest rate. The number of deaths per year over the 10 years from 1995 to 2005 increased by a mean of just 0.3% pa (cumulative), with just 5,200 more deaths in 2005 than 1995 [44]. Strategies to promote "healthy aging" [45] may further reduce the risk of chronic diseases and associated disability, although there are also contrary influences, including rising rates of obesity.

### Health workforce models

Because of market failure in health care and the health workforce [35,46], the market will not arrive at an optimal health workforce solution meaning a clear role for health workforce planning. The improving quality and scope of data collections in the health sector, and linked data sets present the opportunity for more detailed modelling in this area. Tools, such as hardware and software platforms capable of undertaking detailed analysis are increasingly cheap and ubiquitous. The current limitation is in the available models and frameworks that make use of these tools.

**Figure 2:**
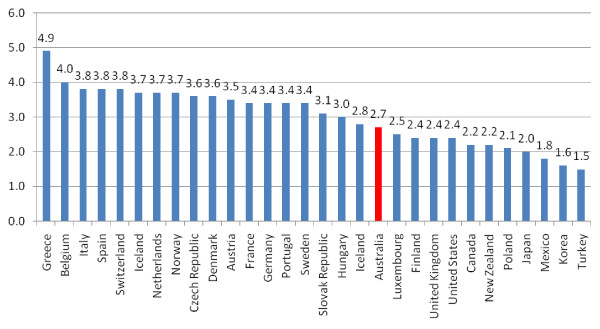
**Practising physicians per 1000 persons, OECD 2005, Source: OECD (2007) **[47].

The dominant approach to 'health workforce planning' has been highly simplistic personnel-to-population ratios. The logic of such approaches is unclear, especially in the face of wide variations in observed ratios over time and place. For instance, the nursing workforce across 28 OECD countries varied in 2002 from 1.7 nurses per 1,000 population in Turkey to 15.3 nurses per 1,000 in Ireland. At 10.4 practising nurses per 1000 population, Australia had the 6^th ^highest nursing ratio, higher than the UK at 8.9, US at 7.9, Canada at 9.4 and New Zealand at 9.4. Practising physicians in 2005 ranged from 1.5 to 4.9 per 1000 population across the OECD, with most countries between 2 and 4, with Australia at 2.7 per 1000 population. (See Figure 2) [47].

O'Brien-Pallas & colleagues in their international review of various health workforce models [48] are most critical of the ratio-based methods of service provision and the associated increase in workforce numbers in line with aggregate population levels. This hardly fits in with the evidence-based approach increasingly demanded in other aspects of health care or health services research.

### Classification of approaches

There have been a number of attempts, internationally, to classify the approaches to modelling health workforce demand. Segal and Robertson [49] described five distinct health workforce forecasting methods:-

1. Historic allocation – commonly the personnel-to-population method as described above.

2. Budget driven mechanisms – also described as a pragmatic approach, where service levels and associated workforce levels are determined in the context of defined expenditure targets. This method has also been referred to as the service-demands method [50].

3. Waiting lists – whereby waiting lists are used as an indicator of imbalance between supply and demand, which as noted above is compromised by the impact of supply on observed demand.

4. Professional group planning models – this is similar to a Delphi method of forecasting, where experts in the field – in this case health professionals – develop the roles and responsibilities and determine the appropriate level of supply.

5. Needs Assessment models – presume that health services planning and funding should enable and support patients and citizens to access best practice care (including preventions services). A needs-based model developed by Segal is described in some detail in a recent publication by Segal at al [51]. Need is defined in this model by best practise care and prevention protocols, applied to the health status of the population. This is translated into a health workforce allowing various options for mapping skills and competencies onto occupations. A similar method is described by Ridoutt et al [52] based on work by O'Connor and Van Konkelneberg (1995) [53]. This type of approach offers an objective evidence-based approach to estimating health workforce need.

**Figure 3:**
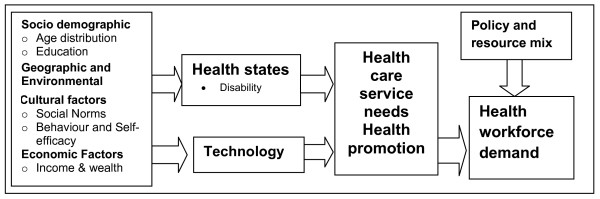
**Factors and pathways influencing health workforce demand**. Source: Zurn et al [1].

Zurn et al [1] have also postulated a framework for analysis of health workforce demand which adopts a needs based framework. They identify the broad influences on population health status – socio-demographic, economic, geographic and cultural, which when combined with available technology, define the health needs of the population. Policy and resource allocation decisions will influence how health needs are translated into health care services and workforce demand (see Figure 3). Health status is shown as mutable, influenced by life style and other factors. This framework is presented as a general schema, rather than a model for estimating health workforce demand. Translation of the schema into a demand model would be highly complex, but also highlights the challenging nature of this task.

Economists such as Maynard [54] also highlighted some of the forecasting challenges of health workforce planning and especially the possible impact of changes in productivity and associated new workforce roles. Changes in technology are likely to affect both how services will be delivered but also health status and disease profile. This, together with changing community expectations – for instance about the role for prevention – will reduce the demand for certain services and associated health professionals and increase demand for others. Policy choices, around matters such as specialisation or multi-skilling in the health workforce will also influence how health service demands are translated into health workforce needs. For instance, the shift in disease profile towards chronic disease and community-based approaches to prevention and management means a greater demand for health professionals with expertise to support lifestyle change such as community and clinical dieticians, exercise physiologists, educators (public health and clinical), health promotion professionals and the possible role for new specialisations. Such changes tend to be gradual and easy to identify. They should be capable of being incorporated into health workforce models. The 'needs-based' demand model developed by Segal has been devised precisely to incorporate changing circumstances.

It is also important as noted by the World Health Organization [5] that health workforce planning consider the need for managers/health planners/researchers as well as the clinical workforce.

## Summary

Market failure in the health care labour market and an apparent mismatch between health workforce supply and demand (defined by need) underline the importance of effective health workforce planning.

The health workforce market is highly complex and influenced by many factors. The changing age distribution of the population is unlikely to have a dominant or critical impact on either health workforce supply or demand despite predictions of some commentators.

Health workforce supply is found to be highly responsive to policy initiatives. This means that if we can establish the level and mix of health workers that will best meet the needs of the community, policies can be devised to adjust supply to that level. Establishing the desirable level and mix of the health workforce should therefore by a primary focus of health workforce research. Health workforce supply strategies can then be devised to meet this defined need.

Developing a planning model that provide a rigorous means of estimating health service demands and an associated health workforce is critical for effective health workforce planning. More research into supply-side issues (worker training, recruitment, retention etc.), will fail to deliver what is achievable in terms of better health, if it proceeds without a complementary and rigorous analysis of demand. Simple population-based ratio methods of estimating workforce demand are demonstrably inadequate. Even supposing such ratios were correct at some point in time, there is no mechanism to adjust for changing community expectations, disease profile, etc.. Given access to high quality data and sound modelling techniques, there is no reason to delay the adoption of sophisticated approaches to modelling health workforce demand.

We argue for an evidenced-based approach to estimating health workforce demand that builds on population health status and an understanding of best practice cost-effective care and prevention, but also one that can model alternative technologies for service delivery. Such a model is currently being trialled in South Australia.

## Competing interests

The authors declare that they have no competing interests.

## Authors' contributions

LS developed the conceptual framework and thesis of the paper; LS and TB jointly drafted the manuscript.
